# Pleiotropy of *FRIGIDA* enhances the potential for multivariate adaptation

**DOI:** 10.1098/rspb.2013.1043

**Published:** 2013-07-22

**Authors:** John T. Lovell, Thomas E. Juenger, Scott D. Michaels, Jesse R. Lasky, Alexander Platt, James H. Richards, Xuhong Yu, Hsien M. Easlon, Saunak Sen, John K. McKay

**Affiliations:** 1 Graduate Degree Program in Ecology, Colorado State University, Fort Collins, CO, USA; 2 Department of Bioagricultural Sciences and Pest Management, Colorado State University, Fort Collins, CO, USA; 3 Section of Integrative Biology, University of Texas at Austin, Austin, TX, USA; 4 Department of Biology, Indiana University, Bloomington, IN, USA; 5 Department of Ecology and Evolutionary Biology and Interdepartmental Program on Bioinformatics, University of California, Los Angeles, CA, USA; 6 Department of Land, Air and Water Resources, University of California, Davis, CA, USA; 7 Department of Epidemiology and Biostatistics, University of California San Francisco, San Francisco, CA, USA

**Keywords:** drought, *Arabidopsis thaliana*, water use efficiency, flowering time

## Abstract

An evolutionary response to selection requires genetic variation; however, even if it exists, then the genetic details of the variation can constrain adaptation. In the simplest case, unlinked loci and uncorrelated phenotypes respond directly to multivariate selection and permit unrestricted paths to adaptive peaks. By contrast, ‘antagonistic’ pleiotropic loci may constrain adaptation by affecting variation of many traits and limiting the direction of trait correlations to vectors that are not favoured by selection. However, certain pleiotropic configurations may improve the conditions for adaptive evolution. Here, we present evidence that the *Arabidopsis thaliana* gene *FRI* (*FRIGIDA*) exhibits ‘adaptive’ pleiotropy, producing trait correlations along an axis that results in two adaptive strategies. Derived, low expression *FRI* alleles confer a ‘drought escape’ strategy owing to fast growth, low water use efficiency and early flowering. By contrast, a dehydration avoidance strategy is conferred by the ancestral phenotype of late flowering, slow growth and efficient water use during photosynthesis. The dehydration avoidant phenotype was recovered when genotypes with null *FRI* alleles were transformed with functional alleles. Our findings indicate that the well-documented effects of *FRI* on phenology result from differences in physiology, not only a simple developmental switch.

## 1. Introduction

Populations of a species are frequently distributed across climatic gradients, where natural selection can lead to adaptation to local conditions. The environmental conditions that cause local adaptation have been well documented through reciprocal transplants and studies of clines [1–6]. These experiments show that divergent patterns of selection cause shifts in the mean values of many traits leading to a multivariate response. Such a response to selection improves fitness and promotes successful adaptation to local conditions. Despite a large body of research, it remains a challenge to determine the specific genetic loci that respond to selection and confer local adaptation [7–10].

Long-term breeding programmes and quantitative genetic studies have demonstrated variation in nearly all traits, and thus a simple lack of additive genetic variation is not expected to constrain adaptation [11]. Instead, a limited amount of genetic variation along vectors of selection has been shown to limit adaptive evolution [12–14]. Theoretically, independence of all loci and phenotypes will improve the potential for adaptation by optimizing evolvability [15] and the response to selection (R) [14,16,17]. However, certain genetic correlations can disrupt the optimal genetic architecture by reducing the amount of genetic variation which is available to selection and causing correlated responses of non-adaptive traits [12,18]. Although this maladaptive role for genetic correlations is not universal [19], genetic correlations may affect R by limiting the dimensionality of the genetic (co)variance matrix or restricting genetic variation to vectors which are not aligned with selection [12–13,18,20].

The combined effects of the pleiotropic loci, which cause genetic correlations, may have a profound impact on patterns of local adaptation. As pleiotropy can constrain multivariate adaptation and cause correlated evolution of adaptive and deleterious phenotypic values, these loci are typically considered ‘antagonistic’ [21–25]. Adaptation is especially constrained when pleiotropic gene action limits phenotypic correlations along a vector orthogonal to that of selection and reduces R [13]. Antagonistic pleiotropy is well documented and has led to the belief that all pleiotropy is maladaptive [26]. However, recent theoretical work has countered this viewpoint by demonstrating that intermediate levels of pleiotropy may actually improve the conditions for adaptation and evolution of complexity [27–29].

To study the adaptive value of pleiotropic loci, it is necessary to assess the effects of genetic variation on the structure of many phenotypes which are subject to correlational selection in nature. Adaptation to drought in plants provides an ideal system to achieve this goal [30–33]. In natural and agricultural systems, annual plants can be adapted to local drought conditions by either growing and reproducing before the onset of drought (drought escape) [31,34–36] or by delaying reproduction, increasing water use efficiency (WUE) and conserving resources (dehydration avoidance) [37–39]. For example, accessions which exhibit early-flowering time (FT) and low WUE were selected for in consistently wet soil and late-season drought conditions [36,40], whereas direct selection on increased WUE favoured a dehydration avoidance strategy in environments with early-season drought [41]. Therefore, adaptation to different local soil moisture conditions and seasonal rainfall patterns contributes to the observed strong correlations between FT, growth rate and WUE within and among species [6,32,38,41–43]. Several studies have suggested that pleiotropy may also affect this correlation [38,44,45].

Here, we provide empirical evidence for an adaptive role of pleiotropy. Using genome-wide approaches, allelic variants and transgenic manipulation, we demonstrate that the ‘FT’ gene, *FRIGIDA* (*FRI*) pleiotropically affects phenotypic variation in growth rate, WUE and FT. Derived, null *FRI* alleles produce a drought escape phenotype (decreased WUE, increased growth rate, decreased FT) relative to the ancestral adaptive strategy. This phenomenon, which we term ‘adaptive pleiotropy’, enhances the likelihood of adaptation by increasing adaptive responses to selection.

## 2. Methods

### (a) *Arabidopsis* *thaliana* genetic resources

We used four sets of genetic variants: TK RILs, a panel of 317 physiologically diverse *A. thaliana* accessions, a nearly isogenic line (FRI-NIL) and *FRI* transgenic overexpression lines (tr-FRI). The TK RILs are the product of a bi-directional cross between two physiologically divergent accessions: TSU-1 (low WUE, short FT) and KAS-1 (high WUE, long FT) [46]. The TK RILs mapping population consists of 343 F9 lines each genotyped at 166 genomic loci. In addition to the published loci, all RILs were genotyped at *FRI* via fragment analysis of PCR product generated across the promoter (primer F: 5′-AGTACTCACAAGTCACAAC-3′; primer R: 5′-GAAGATCATCGAATTGGC-3′) [47]. The 317 accession panel was genotyped at this marker (FRIdel1) and two additional markers: FRIdel2 (primer F: 5′-AGATTTGCTGGATTTGATAAGG-3′; primer R: 5′-ATATTTGATGTGCTCTCC-3′) and FRIcap (primer F: 5′-CCATAGACGAATTAGCTGC-3′; primer R: 5′-AGACTCCAGTATAAGAAG-3′). The 317 accessions and TK RILs are listed in the electronic supplementary material, tables S1 and S2 and are available from the *Arabidopsis* stock center (http://www.arabidopsis.org/).

The FRI-NIL was generated by introgressing a functional *FRI* allele from the Sf-2 line into wild-type (WT) Col-0, the reference *A. thaliana* accession with a null *FRI* allele [47–49]. The tr-FRI transgenic over expressed line was generated by ligating FRI-GFP into the *Xma*I and *Xho*I sites of 35SpBARN vector and then transformed a into Col-0 background using the floral dip method. We used only *FRI* transgenic lines that exhibited a late flowering phenotype. The FRI-NIL and the transgenic line ‘FRI-GFP Col T2 #20’ are available from S.D.M. and X.Y. We also present WUE data from FRI-NIL and Columbia genotypes with knocked-out *FLC* alleles. See Michaels & Amasino [50] for details on these lines.

### (b) Plant growth and phenotypic analysis

Phenotypic analyses of the TK RILs, the FRI-NIL, tr-FRI and Col-0 were conducted in a Conviron ATC60 growth chamber (Controlled Environments, Winnipeg, MB, Canada) at Colorado State University (CSU). All plants were grown at 12 h, 40 per cent humidity, 23°C days and 12 h, 50 per cent humidity, 18°C nights. Photosynthetic photon flux density during daylight was approximately 330 μmol m^−2^ s^−1^. All plants except those analysed for gas exchange were grown in 2′ plastic pots containing Fafard 4p mix (Conrad Fafard Inc. Agawam, MA). Gas exchange measurements were taken on plants grown in the same conditions in modified Cone-tainer pots (Stuewe and Sons, Tangent, OR). A 195-line subset of the 317 line panel was grown at University of Texas, Austin in promix BT potting soil and 164 ml Cone-tainer pots under long-day photoperiod conditions (16 L : 8 D) at approximately 18–21°C. Consistent with previous studies, these long-day environmental conditions induced flowering much more quickly than the 12/12 h conditions at CSU.

Gas exchange physiology was measured with an LI-6400 photosynthesis system (LiCor Inc, Lincoln, NB) equipped with a custom whole-plant gas exchange cuvette. A total of 20 measurements were taken over a 2 min period for each of 20 plants (10 replicates/genotype) at two time points (14 and 21 days post-germination). The photosynthetic parameters (*A, c*_i_ and *g*_s_) were estimated following von Caemmerer & Farquhar [51]. Gas exchange data were analysed in a mixed-model framework where genotype was fixed, and measurement and date were nested within individual as a random effect in JMP Genomics v. 5.0 (SAS Institute, Cary, NC). We also generated *A*/*c*_i_ curves by measuring photosynthetic rate across nine levels of external CO_2_ concentrations using a different set of plants grown hydroponically. We compared *A* between the FRI-NIL and Col-0 controlling for variation in *c*_i_ with a mixed effect ANOVA. The genotype was a fixed effect, and *c*_i_ was a continuous, random covariate.

We measured WUE, growth rate and FT for each plant (*n*/genotype = 10). Flowering initiation was recorded when a visible bolting structure first appeared at the apical meristems; FT is calculated as the number of days between germination and initiation of flowering. We analysed carbon isotope composition (*δ*^13^C), a surrogate measure of WUE [38,52], on lyophilized, finely ground rosette leaves at the Stable Isotope Facility at University of California, Davis (UCD; http://stableisotopefacility.ucdavis.edu/). Leaves were harvested before the onset of flowering of the earliest accession at a single time-point for all lines. Using images taken directly over the rosette, we assessed leaf area in ImageJ (http://rsbweb.nih.gov/ij/). These data were used to calculate relative growth rate of leaf area (GR_la_ = [ln(LA*_t_*_2_) – ln(LA*_t_*_1_)]/(*t*_2_ − *t*_1_)), where LA is leaf area at time 1 (*t*_1_) and time 2 (*t*_2_). We analysed the effect of genotype on WUE, FT and GR_la_ via one-way ANOVA in JMP Genomics v. 5.0.

### (c) Quantitative trait locus analysis

We analysed quantitative trait loci (QTL) for WUE and FT in R/qtl [53] using the following settings: (i) imputations (256 draws) to generate a complete and even genome-wide pseudo-marker grid of 2 cM for mapping, (ii) 10 000 permutations to calculate QTL incorporation thresholds at an experiment wise *α* = 0.05, (iii) stepwise model selection scanning for epistatic and additive QTL at each step [54], (iv) iterative position refinement analysis by holding all but one QTL constant and varying the position of the focal QTL and re-calculating the penalized logarithm (base 10) of the odds (LOD) score for the model and (v) fitting the refined model via ANOVA to calculate the effect size, per cent variance explained and LOD score for each QTL. The allelic effect at *FRI* was compared via one-way ANOVA in JMP Genomics v. 5.0. To further refine the bi-phenotype QTL position, we standardized the LOD scores by the largest value for each phenotype (stand. LOD) and summed the bivariate scores for each point on the genotype grid, then calculated the bivariate QTL interval as the point where the summed LOD scores decreased to the average single phenotype odds ratio at a given map position.

### (d) Quantification of the FRI-NIL Sf-2 introgression

Whole genome sequence was obtained by paired-end Illumina sequencing at the UCD Genome Center (http://www.genomecenter.ucdavis.edu/). A reference-based assembly of the TAIR 9 Columbia genome was conducted in shore (http://1001genomes.org/software/shore.html) to call single nucleotide polymorphisms (SNPs) [55] and identify the size of the introgression.

### (e) Gene expression analysis

Genome-wide gene expression was determined via Affymetrix (Affymetrix Inc. Santa Clara, CA) AthSNPtile arrays for all TK RILs. We screened for all genes within 50 kb of the QTL point estimate (CH_4_ 237 060–337 060 bp) and compared expression levels between TSU-1 and KAS-1 alleles at each gene, then corrected for multiple comparisons via *q*-value calculations (R package qvalue http://www.bioconductor.org/packages/release/bioc/html/qvalue.html).

### (f) Analysis of population structure at *FRIGIDA*

We conducted three separate population genetic analyses using the publicly available genome-wide SNP data (http://cynin.gmi.oeaw.ac.at/home/resources/atpolydb) [56,57]. We imputed the functionality of *FRI* for all lines by extracting all SNPs within 100 kb of *FRI* and training a classification model, support vector machines (SVMs) with a radial basis function [58], using data on SNPs and *FRI* functionality for the 317 genotypes in our panel that had both SNP and *FRI* data. After a grid search of tuning parameter values, our final SVM model predicted *FRI* functionality with 95 per cent accuracy in fourfold cross-validation. We tested the accuracy of the SVM model using *n*-fold cross-validation: after selecting *n* accessions at random, we tested the accuracy of SVM models in *n*-fold cross-validation (i.e. leave-one-out cross-validation) for *n* = 10, 15, 20, 25, 30, 35 and 40. For each value of *n*, we cross-validated the SVM predictions for 20 random subsets of accessions. For *n* = 10, SVM models were on average 87 per cent accurate in cross-validation. By *n* = 20, models were 93 per cent accurate in cross-validation. This signifies that the SNP associations with *FRI* functionality are easily observable in even small samples of accessions. Using this model, we then imputed the allelic state (binned into functional or null categories) for all accessions in the SNP database.

We calculated genome-wide *F*_ST_ in plink [59] by classifying the accessions as ‘functional’ or ‘non-functional’ *FRI* and calculating the molecular variance between and within these allele classes. We generated 5000 random divisions at the same frequency as the *FRI* allele classes. These permutations allow us to assess the significance of the *F*_ST_ measure compared with random evolution. We conducted two additional analyses with subsets of the available accessions. Ten of the 574 sites sampled by Horton *et al* [57] showed within-population variation at *FRI*. Using these populations and geographical clusters at the country level [57], we calculated an average heterozygosity over SNPs sampled at 50 kb intervals (*H_t_*). Then, we split the population based on *FRI* phenotype, calculated an average heterozygosity within each subpopulation (*H*_s_) in the same way and took the mean of those. We used these *H_t_* and *H*_s_ values to calculate genome-wide *F*_ST_ based on the *FRI* phenotype. We bootstrapped to calculate significance by dividing the data at a random subset of 5000 SNPs with similar frequency to *FRI* and recalculating *F*_ST_.

### (g) Comparison of climatic variables associated with *FRI* variation

*FRI* functionality calls, latitude and longitude for each line were input into DIVA-GIS (www.diva-gis.org). The 19 BIOCLIM (www.bioclim.org) climatic variables were extracted for each point. *FRI* allelic association with these variables was made via *t*-tests with significance corrected for multiple comparisons by Bonferroni adjustments. The distribution of the climate under each allele was compared by ranking the climate variables and plotting the relative position of each allele relative to its rank.

## 3. Results and discussion

### (a) Mapping the water use efficiency–flowering time correlation

**Figure 1.**
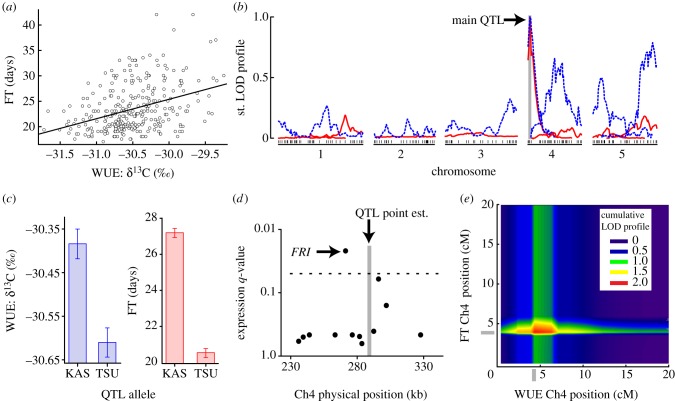
One QTL affects both WUE and FT and is associated with functionally divergent *FRI* alleles. (*a*) The WUE–FT correlation observed in nature is present within our recombinant mapping population; bivariate breeding values for each TK RIL (hollow points) and the linear model (solid line) are plotted. (*b*) Standardized LOD profile scores for both WUE (dashed blue) and FT (solid red) colocalize at 4 cM on chromosome 4. (*c*) WUE and FT of each RIL are split by genotype and plotted with means ± s.e. of the mean. (*d*) The only significant expression difference within 100 kb of the QTL point estimate (chromosome 4287.06 kb) is at *FRI* (labelled). Horizontal line: FDR = 0.05. (*e*) Precise co-localization of the main QTL for WUE and FT is shown: standardized, summed LOD profiles are plotted for each pairwise locus combination across the first 20 cM of Ch4. Grey bars on the axes indicate the point where the maximum score is achieved.

We measured FT and WUE of 195 *A. thaliana* accessions in a common garden. The genetic correlation between WUE and FT is positive and significant: WUE explains nearly 40 per cent of FT variation (*n* = 195, *r*^2^ = 0.395, *p* < 0.0001; electronic supplementary material, figure S1). If this correlation results from many loci independently affecting each phenotype, then recombination between differently adapted lines will break down this favourable correlation. To test the cause of the WUE–FT correlation, we used TK RILs from two phenotypically divergent accessions, TSU-1 (low WUE, short FT) and KAS-1 (high WUE, long FT) [46] (see the electronic supplementary material, figure S1). Experimental crosses induce recombination and break up linkage disequilibrium across these genomes. Despite a large reduction in linkage disequilibrium, FT and WUE remained significantly correlated (*n* = 304, *r*^2^ = 0.138, *p* < 0.0001; figure 1*a*) in the TK RILs, demonstrating that either tight genetic linkage or pleiotropy caused WUE and FT to covary.

To determine the genetic basis of the remaining WUE–FT correlation in the RILs, we conducted a QTL analysis by simultaneously scanning for genomic loci significantly associated with both phenotypes. Stepwise model selection (*α* = 0.05) revealed a total of 11 different QTLs across both traits (see the electronic supplementary material, table S3). Only one QTL was found that affected both phenotypes and this QTL was the largest for each individual trait (figure 1*b* and electronic supplementary material, table S3). Lines with the KAS-1 QTL allele had later FT (d.f. = 1, *F*_338.8_, *p* < 0.0001), and higher WUE (d.f. = 1, *F*_19.14_, *p* < 0.0001) than TSU-1 alleles (figure 1*c*). The genetic correlation between WUE and FT is well documented in agricultural breeding populations and studies of local adaptation in nature [38,44–45]. High WUE decreases photosynthetic assimilation rates and the amount of fixed carbon available for flowering. As the initiation of flowering is affected, in part, by resource availability [60,61], a physiological connection between WUE and FT is plausible.

### (b) Cloning the water use efficiency–flowering time quantitative trait locus

To identify all possible causal variants underlying the main QTL, we re-sequenced both parents and analysed gene expression in the TK RILs for loci within a 100 kb region surrounding the QTL. Within this region, only *FRI* (*FRIGIDA*) is differentially expressed between TSU-1 and KAS-1 (figure 1*d*). Re-sequencing of both parents revealed a 376 bp deletion within the promoter of the TSU-1 *FRI* allele, but a functional allele in KAS-1. We genotyped the *FRI* deletion in all TK RILs (see the electronic supplementary material, table S2). After adding the *FRI* polymorphism to the linkage map, we conducted a multi-trait position refinement analysis. The QTL maps to a single pleiotropic locus at the nearest pseudo-marker to *FRI*: chromosome 4, position 4.0 cM (figure 1*e*).

*FRI* is a particularly good candidate gene underlying the FT QTL. Derived mutations that reduce expression have been involved in the evolution of spring annual types from the ancestral state of a fully functional *FRI* and a winter annual life history [47,62]; allelic variation at *FRI* contributes to variation in FT across diverse accessions [49,63–65]. *FRI* is also a candidate for WUE [38,66]. Biogeographic analyses have associated lines with functional *FRI* alleles, such as KAS-1, to regions with lower precipitation; these environments would favour drought adaptation via dehydration avoidance [6,67,68].

**Figure 2.**
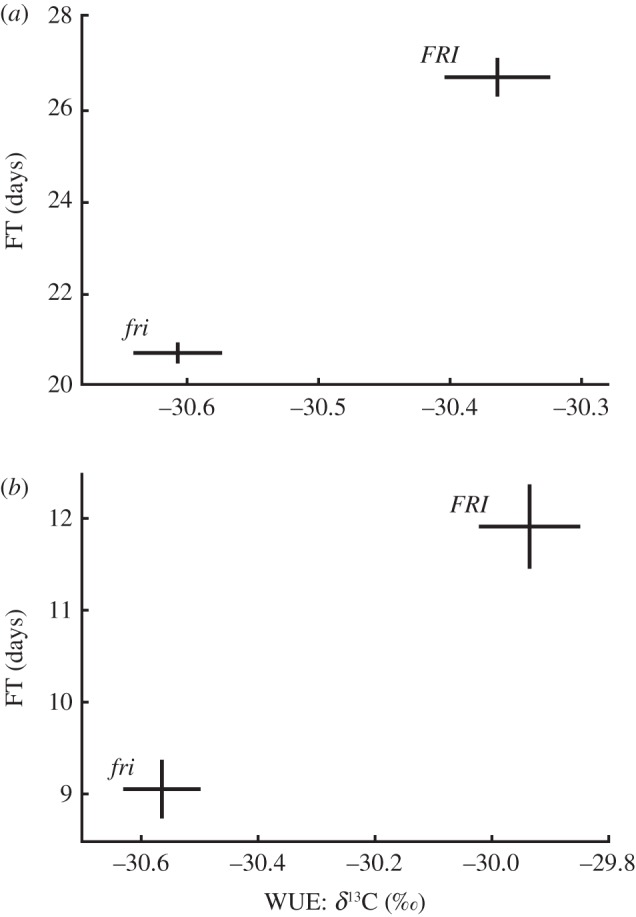
Phenotypic variance of both WUE and FT is due to natural variation at *FRI*. Means ± s.e. are plotted for each allele class (*FRI*, functional; *fri,* null alleles). (*a*) *FRI* functional variation significantly explains WUE and FT variation in the TSU-1xKAS-1 mapping population and (*b*) 195 natural accessions when grown in a common garden.

To further assess the pleiotropic effects of the *FRI* locus, we also genotyped 195 *A. thaliana* accessions at *FRI* to determine functionality (see the electronic supplementary material, table S1). Consistent with pleiotropy and our observation in the TK RILs (figure 2*a*), phenotypic variation in both WUE (ANOVA d.f. = 1, *F*_51.705_, *p* < 0.0001) and FT (ANOVA d.f. = 1, *F*_34.643_, *p* < 0.0001) is predicted by functional variation at *FRI* in natural populations (figure 2*b*). In the 195 accessions, *FRI* explains 30.2 per cent and 24.7 per cent of the total phenotypic variation of WUE and FT, respectively. Interestingly, this ‘FT’ gene explains less phenotypic variation in FT than WUE in wild accessions. Null *FRI* alleles represent a derived state of early FT and lower WUE relative to functional alleles; a drought escape strategy.

### (c) Physiological pleiotropy of *FRI*

**Figure 3.**
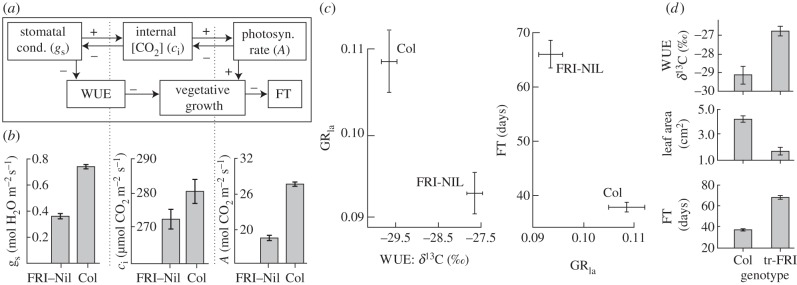
*FRI* pleiotropically affects WUE, GR_la_ and FT. (*a*) The conceptual model demonstrates the mechanism of pleiotropy, upstream variation in gas exchange causes subsequent changes in GR_la_ and FT. (*b*) FRI-NILs show reduced gas exchange compared with WT Col-0. (*c*) FRI-NILs have increased WUE and reduced GRla compared with WT Col-0. (*d*) Transgenic overexpression lines show the same pattern as the FRI-NIL. Least square means ± s.e. are presented in (*b–d*).

To test for the phenotypic effects of *FRI*, we compared the phenotypes of a near isogenic line with a functional *FRI* allele (FRI-NIL) to the wild-type progenitor Col-0 (WT) which contains a null *fri* allele. We phenotyped the three major physiological determinants of WUE: photosynthetic rate (*A*), leaf internal CO_2_ concentration (*c*_i_) and stomatal conductance (*g*_s_). Stomatal conductance, which directly alters leaf water-loss dynamics, also affects *A* by regulating the supply of CO_2_ and thus, *c*_i_. For example, low *g*_s_ reduces *A* by limiting *c*_i_, resulting in increased WUE, decreased growth rate and delayed FT [43] (figure 3*a*).

The FRI-NIL (functional *FRI*) had decreased *g*_s_ (contrast d.f. = 1, *F*_208.48_, *p* < 0.0001), marginally lower *c*_i_ (contrast d.f. = 1, *F*_3.28_, *p* = 0.072) and significantly lower *A* (contrast d.f. = 1, *F*_255.23_, *p* < 0.0001) relative to WT (null *fri*), indicating stomatal limitation to growth through *A* (figure 3*b*). Additionally, WUE (d.f. = 1, *F*_43.14_, *p* < 0.0001), GR_la_ (d.f. = 1, *F*_22.47_, *p* < 0.0001) and FT (d.f. = 1, *F*_125.22_, *p* < 0.0001) all significantly differ between FRI-NIL and WT (figure 3*c*). To determine the physiological mechanism for increased WUE, we modulated *c*_i_ and repeatedly measured *A*. Supporting a decrease in *g*_s_ as the basis for increased WUE, no significant difference in photosynthetic capacity was found between the WT and FRI-NIL while controlling for *c*_i_ (d.f. = 288, *F*_1.701_, *p* = 0.1932; electronic supplementary material, figure S2).

Many functional analyses have found that *FRI* produces a transcription factor that induces expression of *FLC*, inhibiting floral development [50,62,63,65,69,70]. To place our analyses in the context of these results, we analysed WUE for WT and FRI-NIL lines which have knocked-out *FLC* alleles. Consistent with the epistasis observed to affect FT, *FRI* confers increased WUE only in the presence of a functional *FLC* (contrast d.f. = 1, *F*_44.77_, *p* < 0.0001), but not the when associated with a null *flc* allele (contrast d.f. = 1, *F*_0.79_, *p* = 0.38; electronic supplementary material, figure S3).

The FRI-NIL (also referred to as ‘Sf-2 *FRI* in Col’ or ‘Col-*FRI*’) is one of the most used genetic resources in the FT literature [38,49,71]. These studies assume that the FRI-NIL carries a single, narrow, introgression of the Sf-2 genome which contains a functional *FRI* allele; however, this assumption has never been tested. To assess the size of the Sf-2 introgression, we re-sequenced the FRI-NIL, aligned the reads to the TAIR 9 Columbia genome, called SNPs and mapped SNP density to the reference genome. Many SNPs exist between Sf-2 and Col-0 (data are publically available at http://mus.well.ox.ac.uk/19genomes [72]). High SNP density between the FRI-NIL and Col-0 exists solely on proximate Chr. 4 (see the electronic supplementary material, figure S4*a,b*). The region of elevated SNP density represents a single 1.070 Mb (±10 kb) Sf-2 introgression that contains *FRI* as well as the other 325 gene models between AT4G00005 and AT4G02710. Although most studies that use the FRI-NIL assume the only genotypic divergence exists at *FRI*, this is obviously not the case.

To unambiguously determine whether the effects observed in the FRI-NIL were due to *FRI*, we compared WUE, FT and GR_la_ between WT Col-0 and transgenic lines (Col-0 overexpressing *FRI*: tr-FRI). Under well-watered conditions, tr-FRI had greater WUE (d.f. = 1, *F*_57.25_, *p* < 0.0001), decreased GR_la_ (d.f. = 1, *F*_22.32_, *p* < 0.0001) and later FT (d.f. = 1, *F*_179.1_, *p* < 0.0001) than WT (figure 3*d*). As *FRI* functionality is the only DNA sequence difference between these lines, *FRI is pleiotropic and controls covariation of three traits along a vector shown to be adaptive*. Our conclusion is supported by QTL, natural accession, NIL and transgenic comparisons.

### (d) The population genetics of adaptive pleiotropy

Population genetic models are at odds about the role of pleiotropy in maintaining variation within and among populations. Pleiotropic gene action may cause non-adaptive and adaptive phenotypes to covary, thus reducing the efficacy of correlational selection and permitting the persistence of multiple allelic states within populations [73]. However, where the effects of pleiotropy are more aligned with the direction of selection, within-population variation can be purged by strong directional selection [74]. Therefore, we predicted low levels of within-population variation at *FRI*, because multivariate selection would favour either a functional (drier habitats) or non-functional allele (wetter habitats). In addition, if variation at *FRI* can lead to local adaptation, then we predicted increased population structure (across the entire genome) between functional and non-functional *FRI* classes.

A population genetic test for adaptive pleiotropy is complicated in our study as *FRI* may cause population structure through both adaptive pleiotropy and allochrony: FRI-NILs and tr-FRI lines flowered at least 28 and 32 days later than Col-0, respectively. All main-raceme Col-0 flowers had been pollinated and produced fruits before any FRI-NIL or tr-FRI lines produced open flowers. In the greenhouse environment, single mutations at *FRI* can produce a reproductive isolation index near 1.0. However, assortative mating owing to variation at *FRI* may be tempered in nature as the environment has a profound effect on phenology [71].

To test for evidence of reproductive isolation between accessions and populations that differ at *FRI*, we first imputed *FRI* functionality of 1188 accessions [57], then compared the group of individuals with derived, weak alleles (i.e. null Col-0 missense and Ler deletion alleles) to the group of individuals with functional, ancestral-type *FRI*. We then calculated *F*_ST_ between *FRI* allele functional classes in plink [59]. *F*_ST_ values averaged across 216 130 SNPs are significantly greater between the *FRI* functionality classes than is expected from genome-wide subsampling (*p* < 0.0001; electronic supplementary material, figure S5). To control for geographical population structure, we divided the global sample into 11 geographical regions according to Horton *et al.* [57]. Ten of 11 geographical regions showed elevated *F*_ST_ at *FRI* compared with a genome-wide sample of sites with the same allele frequencies as the *FRI* functional variants (see the electronic supplementary material, table S5). These results show that elevated global *F*_ST_ when sorting by *FRI* is due to a lack of within-population variation in *FRI*. Less than 2 per cent of 574 local populations harboured functional variants at *FRI*.

While extremely low within-population variation is present at *FRI*, functionally divergent alleles have gone to fixation in geographically proximate populations. Several authors have shown that an abundance of derived null *FRI* alleles are present in nature, far more than would be expected by chance [75,76]. Here, we demonstrate that these mutations cause a phenotypic leap between drought adaptation strategies which may promote adaptation to novel ecological conditions. Combined, the strong signature of selection, high levels of population structure and lack of within-population variation observed at *FRI* suggests an adaptive role of this pleiotropy.

### (e) *FRI* and drought adaptation

Previous studies have found that the early flowering, low WUE phenotypes associated with drought escape are adaptive in sites without consistent low soil moisture [36]. Although we did not directly measure selection in this study, we used the large body of work on drought adaptation to infer the adaptive value of specific trait combinations. We predicted that due to the drought escape strategy conferred by derived loss of function mutations at *FRI*, accessions with these alleles would inhabit environments with consistently wetter growing seasons, relative to accessions with functional *FRI* alleles. To confirm the allelic association with drought, we generated a climate envelope for both *FRI* allele classes (see the electronic supplementary material, table S5). Functional alleles tend to be present in areas with lower growing season precipitation than non-functional alleles (*t* = −3.68, *p* = 0.0003; electronic supplementary material, figure S6).

We have demonstrated that lines that diverged only at *FRI* exhibit altered positions along an adaptive phenotypic correlation. Scarcelli *et al*. [25] found antagonism between the floral morphology traits affected by *FRI*, and we cannot rule out that a portion of *FRI*'s pleiotropic gene action is maladaptive. However, analyses presented here demonstrate a strong adaptive role of the physiological and phenological phenotypic correlations conferred by *FRI*. Given our results, it is not surprising that *FRI* is associated with strong population genetic signatures of diversifying selection [65,75,76]. Studies demonstrating historical selection on *FRI* invoke the timing of flowering as the phenotype under selection [65]. Our results indicate that the observed signature of selection is not only an effect of FT variation, but may also be due to upstream physiological effects.

## 4. Conclusions

We have presented a mechanistic understanding of how *FRI* alters physiology, phenology and confers local adaptation. Phenology, growth rate and water-use physiology have been mapped to similar genomic loci or correlated in natural or experimental populations [31,38,46,52,66]. Here, we have demonstrated that *FRI* causes these adaptive correlations to be heritable. Although we present a situation where pleiotropy controls phenotypic variation along a vector known to be adaptive, we have not measured the efficacy of or response to selection in the field. Fitness measures in diverse common gardens with watering treatments would allow for direct inference of the adaptive value of *FRI*.

To date, most gene annotation and characterization is conducted by forward or reverse genetics whereby a single gene or trait is under consideration. Our results indicate that a more holistic approach to phenotyping and whole plant, integrative approaches for annotating gene function may reveal complex patterns of pleiotropy among ecologically correlated phenotypes. It is possible that many trait associations are not purely a product of correlational selection, but also affected by adaptive pleiotropy.

## References

[1] Agren J Schemske DW 2012 Reciprocal transplants demonstrate strong adaptive differentiation of the model organism *Arabidopsis thaliana* in its native range. New Phytol. 194, 1112–1122. 10.1111/j.1469-8137.2012.04112.x (doi:10.1111/j.1469-8137.2012.04112.x) 22432639

[2] Hall MC Willis JH 2006 Divergent selection on flowering time contributes to local adaptation in *Mimulus guttatus* populations. Evolution 60, 2466–2477. 17263109

[3] Huey RB Gilchrist GW Carlson ML Berrigan D Serra L 2000 Rapid evolution of a geographic cline in size in an introduced fly. Science 287, 308–309. 10.1126/science.287.5451.308 (doi:10.1126/science.287.5451.308) 10634786

[4] Moore JS Hendry AP 2005 Both selection and gene flow are necessary to explain adaptive divergence: evidence from clinal variation in stream stickleback. Evol. Ecol. Res. 7, 871–886.

[5] Reznick DN Ghalambor CK 2001 The population ecology of contemporary adaptations: what empirical studies reveal about the conditions that promote adaptive evolution. Genetica 112, 183–198. 10.1023/A:1013352109042 (doi:10.1023/A:1013352109042) 11838765

[6] Stinchcombe JR Weinig C Ungerer M Olsen KM Mays C Halldorsdottir SS Purugganan MD Schmitt J 2004 A latitudinal cline in flowering time in *Arabidopsis thaliana* modulated by the flowering time gene FRIGIDA. Proc. Natl Acad. Sci. USA 101, 4712–4717. 10.1073/pnas.0306401101 (doi:10.1073/pnas.0306401101) 15070783 PMC384812

[7] Lande R 1984 The genetic correlation between characters maintained by selection, linkage and inbreeding. Genet. Res. 44, 309–320. 10.1017/S0016672300026549 (doi:10.1017/S0016672300026549) 6530140

[8] Lande R Arnold SJ 1983 The measurement of selection on correlated characters. Evolution 37, 1210–1226. 10.2307/2408842 (doi:10.2307/2408842) 28556011

[9] Rockman MV 2012 The QTN program and the alleles that matter for evolution: all that's gold does not glitter. Evolution 66, 1–17. 10.1111/j.1558-5646.2011.01486.x (doi:10.1111/j.1558-5646.2011.01486.x) 22220860 PMC3386609

[10] Orr HA 1998 The population genetics of adaptation: the distribution of factors fixed during adaptive evolution. Evolution 52, 935–949. 10.2307/2411226 (doi:10.2307/2411226) 28565213

[11] Lynch M Walsh B 1998 Genetics and analysis of quantitative traits. Sunderland, MA: Sinauer.

[12] Kirkpatrick M 2009 Patterns of quantitative genetic variation in multiple dimensions. Genetica 136, 271–284. 10.1007/s10709-008-9302-6 (doi:10.1007/s10709-008-9302-6). 18695991

[13] Etterson JR Shaw RG 2001 Constraint to adaptive evolution in response to global warming. Science 294, 151–154. 10.1126/science.1063656 (doi:10.1126/science.1063656) 11588260

[14] Schluter D 1996 Adaptive radiation along genetic lines of least resistance. Evolution 50, 1766–1774. 10.2307/2410734 (doi:10.2307/2410734) 28565589

[15] Wagner GP Altenberg L 1996 Perspective: complex adaptations and the evolution of evolvability. Evolution 50, 967–976. 10.2307/2410639 (doi:10.2307/2410639) 28565291

[16] Blows MW Hoffmann AA 2005 A reassessment of genetic limits to evolutionary change. Ecology 86, 1371–1384. 10.1890/04-1209 (doi:10.1890/04-1209)

[17] Lande R 1982 A quantitative genetic theory of life-history evolution. Ecology 63, 607–615. 10.2307/1936778 (doi:10.2307/1936778)

[18] MitchellOlds T 1996 Pleiotropy causes long-term genetic constraints on life-history evolution in *Brassica rapa*. Evolution 50, 1849–1858. 10.2307/2410742 (doi:10.2307/2410742) 28565577

[19] Agrawal AF Stinchcombe JR 2009 How much do genetic covariances alter the rate of adaptation? Proc. R. Soc. B 276, 1183–1191. 10.1098/rspb.2008.1671 (doi:10.1098/rspb.2008.1671) PMC2679087 19129097

[20] Blows MW Chenoweth SF Hine E 2004 Orientation of the genetic variance-covariance matrix and the fitness surface for multiple male sexually selected traits. Am. Nat. 163, 329–340. 10.1086/381941 (doi:10.1086/381941) 15026971

[21] Curtsinger JW Service PM Prout T 1994 Antagonistic pleiotropy, reversal of dominance, and genetic-polymorphism. Am. Nat. 144, 210–228. 10.1086/285671 (doi:10.1086/285671)

[22] Hedrick PW 1999 Antagonistic pleiotropy and genetic polymorphism: a perspective. Heredity 82, 126–133. 10.1038/sj.hdy.6884400 (doi:10.1038/sj.hdy.6884400)

[23] Houle D 1991 Genetic covariance of fitness correlates: what genetic correlations are made of and why it matters. Evolution 45, 630–648. 10.2307/2409916 (doi:10.2307/2409916) 28568816

[24] Rose MR 1982 Antagonistic pleiotropy, dominance, and genetic variation. Heredity 48, 63–78. 10.1038/hdy.1982.7 (doi:10.1038/hdy.1982.7)

[25] Scarcelli N Cheverud JM Schaal BA Kover PX 2007 Antagonistic pleiotropic effects reduce the potential adaptive value of the FRIGIDA locus. Proc. Natl Acad. Sci. USA 104, 16 986–16 991. 10.1073/pnas.0708209104 (doi:10.1073/pnas.0708209104) PMC2040464 17940010

[26] Otto SP 2004 Two steps forward, one step back: the pleiotropic effects of favoured alleles. Proc. R. Soc. Lond. B 271, 705–714. 10.1098/rspb.2003.2635 (doi:10.1098/rspb.2003.2635) PMC1691650 15209104

[27] Ostman B Hintze A Adami C 2012 Impact of epistasis and pleiotropy on evolutionary adaptation. Proc. R. Soc. B 279, 247–256. 10.1098/rspb.2011.0870 (doi:10.1098/rspb.2011.0870) PMC3223680 21697174

[28] Wang Z Liao B-Y Zhang J 2010 Genomic patterns of pleiotropy and the evolution of complexity. Proc. Natl Acad. Sci. USA (doi:10.1073/pnas.1004666107) 10.1073/pnas.1004666107 PMC2964231 20876104

[29] Hill WG Zhang XS 2012 Assessing pleiotropy and its evolutionary consequences: pleiotropy is not necessarily limited, nor need it hinder the evolution of complexity. Nat. Rev. Genet. 13, 295–295. 10.1038/nrg2949-c1 (doi:10.1038/nrg2949-c1) 22349131

[30] Bray EA 1997 Plant responses to water deficit. Trends Plant Sci. 2, 48–54. 10.1016/S1360-1385(97)82562-9 (doi:10.1016/S1360-1385(97)82562-9)

[31] Meyre D Leonardi A Brisson G Vartanian N 2001 Drought-adaptive mechanisms involved in the escape/tolerance strategies of *Arabidopsis Landsberg erecta* and Columbia ecotypes and their F1 reciprocal progeny. J. Plant Physiol. 158, 1145–1152. 10.1078/S0176-1617(04)70141-8 (doi:10.1078/S0176-1617(04)70141-8)

[32] Sultan SE Bazzaz FA 1993 Phenotypic plasticity in *Polygonum persicaria* 2. Norms of reaction to soil moisture and the maintenance of genetic diversity. Evolution 47, 1032–1049. 10.2307/2409973 (doi:10.2307/2409973) 28564276

[33] Touchette BW Iannacone LR Turner GE Frank AR 2007 Drought tolerance versus drought avoidance: a comparison of plant–water relations in herbaceous wetland plants subjected to water withdrawal and repletion. Wetlands 27, 656–667. 10.1672/0277-5212(2007)27[656:DTVDAA]2.0.CO;2 (doi:10.1672/0277-5212(2007)27[656:DTVDAA]2.0.CO;2)

[34] Araus JL Slafer GA Reynolds MP Royo C 2002 Plant breeding and drought in C-3 cereals: what should we breed for? Ann. Bot. 89, 925–940. 10.1093/aob/mcf049 (doi:10.1093/aob/mcf049) 12102518 PMC4233799

[35] Franks SJ Sim S Weis AE 2007 Rapid evolution of flowering time by an annual plant in response to a climate fluctuation. Proc. Natl Acad. Sci. USA 104, 1278–1282. 10.1073/pnas.0608379104 (doi:10.1073/pnas.0608379104) 17220273 PMC1783115

[36] Sherrard ME Maherali H 2006 The adaptive significance of drought escape in *Avena barbata*, an annual grass. Evolution 60, 2478–2489. 17263110

[37] Donovan LA Dudley SA Rosenthal DM Ludwig F 2007 Phenotypic selection on leaf water use efficiency and related ecophysiological traits for natural populations of desert sunflowers. Oecologia 152, 13–25. 10.1007/s00442-006-0627-5 (doi:10.1007/s00442-006-0627-5) 17165094

[38] McKay JK Richards JH Mitchell-Olds T 2003 Genetics of drought adaptation in *Arabidopsis thaliana.*: I. Pleiotropy contributes to genetic correlations among ecological traits. Mol. Ecol. 12, 1137–1151. 10.1046/j.1365-294X.2003.01833.x (doi:10.1046/j.1365-294X.2003.01833.x) 12694278

[39] Rosenthal DM Stiller V Sperry JS Donovan LA 2010 Contrasting drought tolerance strategies in two desert annuals of hybrid origin. J. Exp. Bot. 61, 2769–2778. 10.1093/jxb/erq109 (doi:10.1093/jxb/erq109) 20435695 PMC2882268

[40] Heschel MS Riginos C 2005 Mechanisms of selection for drought stress tolerance and avoidance in *Impatiens capensis* (Balsaminacea). Am. J. Bot. 92, 37–44. 10.3732/ajb.92.1.37 (doi:10.3732/ajb.92.1.37) 21652382

[41] Heschel MS Donohue K Hausmann N Schmitt J 2002 Population differentiation and natural selection for water-use efficiency in *Impatiens capensis* (Balsaminaceae). Int. J. Plant Sci. 163, 907–912. 10.1086/342519 (doi:10.1086/342519)

[42] Chaves MM Maroco JP Pereira JS 2003 Understanding plant responses to drought: from genes to the whole plant. Funct. Plant Biol. 30, 239–264. 10.1071/fp02076 (doi:10.1071/fp02076) 32689007

[43] Angert AL Huxman TE Barron-Gafford GA Gerst KL Venable DL 2007 Linking growth strategies to long-term population dynamics in a guild of desert annuals. J. Ecol. 95, 321–331. 10.1111/j.1365-2745.2006.01203.x (doi:10.1111/j.1365-2745.2006.01203.x)

[44] Menendez CM Hall AE 1995 Heritability of carbon-isotope discrimination and correlations with earliness in cowpea. Crop Sci. 35, 673–678. 10.2135/cropsci1995.0011183X003500030003x (doi:10.2135/cropsci1995.0011183X003500030003x)

[45] Geber MA Dawson TE 1990 Genetic variation in and covariation between leaf gas-exchange, morphologhy, and development in *Polygonum arenastrum*, an annual plant. Oecologia 85, 153–158. 10.1007/bf00319396 (doi:10.1007/bf00319396) 28312550

[46] McKay JK Richards JH Nemali KS Sen S Mitchell-Olds T Boles S Stahl EA Wayne T Juenger TE 2008 Genetics of drought adaptation in *Arabidopsis thaliana.* II. QTL analysis of a new mapping populations, KAS-1 x TSU-1. Evolution 62, 3014–3026. 10.1111/j.1558-5646.2008.00474.x (doi:10.1111/j.1558-5646.2008.00474.x) 18691264

[47] Johanson U West J Lister C Michaels S Amasino R Dean C 2000 Molecular analysis of FRIGIDA, a major determinant of natural variation in *Arabidopsis* flowering time. Science 290, 344–347. 10.1126/science.290.5490.344 (doi:10.1126/science.290.5490.344) 11030654

[48] Lee I Amasino RM 1995 Effect of vernalization, photoperiod, and light quality on the flowering phenotype of *Arabidopsis* plants containing the *FRIGIDA* gene. Plant Physiol. 108, 157–162. 12228459 10.1104/pp.108.1.157 PMC157316

[49] Lee I Michaels SD Masshardt AS Amasino RM 1994 The late-flowering phenotype of FRIGIDA and mutations in Luminidependens is suppressed in the *Landsberg erecta* strain of *Arabidopsis*. Plant J. 6, 903–909. 10.1046/j.1365-313X.1994.6060903.x (doi:10.1046/j.1365-313X.1994.6060903.x)

[50] Michaels SD Amasino RM 1999 Flowering locus C encodes a novel MADS domain protein that acts as a repressor of flowering. Cell Online 11, 949–956. 10.1105/tpc.11.5.949 (doi:10.1105/tpc.11.5.949) PMC144226 10330478

[51] Caemmerer S Farquhar GD 1981 Some relationships between the biochemistry of photosynthesis and the gas exchange of leaves. Planta 153, 376–387. 10.1007/bf00384257 (doi:10.1007/bf00384257) 24276943

[52] Juenger TE McKay JK Hausmann N Keurentjes JJB Sen S Stowe KA Dawson TE Simms EL Richards JH 2005 Identification and characterization of QTL underlying whole-plant physiology in *Arabidopsis thaliana*: *δ*^13^C, stomatal conductance and transpiration efficiency. Plant Cell Environ. 28, 697–708. 10.1111/j.1365-3040.2004.01313.x (doi:10.1111/j.1365-3040.2004.01313.x)

[53] Broman KW Wu H Sen S Churchill GA 2003 R/qtl: QTL mapping in experimental crosses. Bioinformatics 19, 889–890. 10.1093/bioinformatics/btg112 (doi:10.1093/bioinformatics/btg112) 12724300

[54] Manichaikul A Moon JY Sen S Yandell BS Broman KW 2009 A model selection approach for the identification of quantitative trait loci in experimental crosses, allowing epistasis. Genetics 181, 1077–1086. 10.1534/genetics.108.094565 (doi:10.1534/genetics.108.094565) 19104078 PMC2651044

[55] Ossowski S Schneeberger K Clark RM Lanz C Warthmann N Weigel D 2008 Sequencing of natural strains of *Arabidopsis thaliana* with short reads. Genome Res. 18, 2024–2033. 10.1101/gr.080200.108 (doi:10.1101/gr.080200.108) 18818371 PMC2593571

[56] Atwell S 2010 Genome-wide association study of 107 phenotypes in *Arabidopsis thaliana* inbred lines. Nature 465, 627–631. 10.1038/nature08800 (doi:10.1038/nature08800) 20336072 PMC3023908

[57] Horton MW 2012 Genome-wide patterns of genetic variation in worldwide *Arabidopsis thaliana* accessions from the RegMap panel. Nat. Genet. 44, 212–216. 10.1038/ng.1042 (doi:10.1038/ng.1042) 22231484 PMC3267885

[58] Lasky JR Des Marais DL McKay JK Richards JH Juenger TE Keitt TH 2012 Characterizing genomic variation of *Arabidopsis thaliana*: the roles of geography and climate. Mol. Ecol. 21, 5512–5529. 10.1111/j.1365-294X.2012.05709.x (doi:10.1111/j.1365-294X.2012.05709.x) 22857709

[59] Purcell S 2007 PLINK: a toolset for whole-genome association and population-based linkage analysis. Am. J. Hum. Genet. 81, 559–575. 10.1086/519795 (doi:10.1086/519795) 17701901 PMC1950838

[60] Nord EA Shea K Lynch JP 2011 Optimizing reproductive phenology in a two-resource world: a dynamic allocation model of plant growth predicts later reproduction in phosphorus-limited plants. Ann. Bot. 108, 391–404. 10.1093/aob/mcr143 (doi:10.1093/aob/mcr143) 21712299 PMC3143053

[61] Reekie EG Bazzaz FA 1987 Reproductive effort in plants. 3. Effect of reproduction on vegetative activity. Am. Nat. 129, 907–919. 10.1086/284683 (doi:10.1086/284683)

[62] Shindo C Aranzana MJ Lister C Baxter C Nicholls C Nordborg M Dean C 2005 Role of FRIGIDA and flowering locus C in determining variation in flowering time of *Arabidopsis*. Plant Physiol. 138, 1163–1173. 10.1104/pp.105.061309 (doi:10.1104/pp.105.061309) 15908596 PMC1150429

[63] Gazzani S Gendall AR Lister C Dean C 2003 Analysis of the molecular basis of flowering time variation in *Arabidopsis* accessions. Plant Physiol. 132, 1107–1114. 10.1104/pp.103.021212 (doi:10.1104/pp.103.021212) 12805638 PMC167048

[64] Geraldo N Baurle I Kidou S Hu XY Dean C 2009 FRIGIDA delays flowering in *Arabidopsis* via a cotranscriptional mechanism involving direct interaction with the nuclear cap-binding complex. Plant Physiol. 150, 1611–1618. 10.1104/pp.109.137448 (doi:10.1104/pp.109.137448) 19429606 PMC2705036

[65] Korves TM Schmid KJ Caicedo AL Mays C Stinchcombe JR Purugganan MD Schmitt J 2007 Fitness effects associated with the major flowering time gene *FRIGIDA* in *Arabidopsis thaliana* in the field. Am. Nat. 169, E141–E157. 10.1086/513111 (doi:10.1086/513111) 17427127

[66] Christman MA Richards JH McKay JK Stahl EA Juenger TE Donovan LA 2008 Genetic variation in *Arabidopsis thaliana* for night-time leaf conductance. Plant Cell Environ. 31, 1170–1178. 10.1111/j.1365-3040.2008.01833.x (doi:10.1111/j.1365-3040.2008.01833.x) 18510710

[67] Brock MT Stinchcombe JR Weinig C 2009 Indirect effects of FRIGIDA: floral trait (co)variances are altered by seasonally variable abiotic factors associated with flowering time. J. Evol. Biol. 22, 1826–1838. 10.1111/j.1420-9101.2009.01794.x (doi:10.1111/j.1420-9101.2009.01794.x) 19583697

[68] Mendez-Vigo B Pico FX Ramiro M Martinez-Zapater JM Alonso-Blanco C 2011 Altitudinal and climatic adaptation is mediated by flowering traits and FRI, FLC, and PHYC Genes in *Arabidopsis*. Plant Physiol. 157, 1942–1955. 10.1104/pp.111.183426 (doi:10.1104/pp.111.183426) 21988878 PMC3327218

[69] Michaels SD Amasino RM 2001 Loss of flowering locus C activity eliminates the late-flowering phenotype of FRIGIDA and autonomous pathway mutations but not responsiveness to vernalization. Plant Cell 13, 935–941. 11283346 10.1105/tpc.13.4.935 PMC135534

[70] Caicedo AL Stinchcombe JR Olsen KM Schmitt J Purugganan MD 2004 Epistatic interaction between *Arabidopsis* FRI and FLC flowering time genes generates a latitudinal cline in a life history trait. Proc. Natl Acad. Sci. USA 101, 15 670–15 675. 10.1073/pnas.0406232101 (doi:10.1073/pnas.0406232101) PMC524852 15505218

[71] Wilczek AM 2009 Effects of genetic perturbation on seasonal life history plasticity. Science 323, 930–934. 10.1126/science.1165826 (doi:10.1126/science.1165826) 19150810

[72] Gan X 2011 Multiple reference genomes and transcriptomes for *Arabidopsis thaliana*. Nature 477, 419–423. 10.1038/nature10414 (doi:10.1038/nature10414) 21874022 PMC4856438

[73] Zhang XS Hill WG 2003 Multivariate stabilizing selection and pleiotropy in the maintenance of quantitative genetic variation. Evolution 57, 1761–1775. 10.1554/02-587 (doi:10.1554/02-587) 14503618

[74] Waxman D Peck JR 1998 Pleiotropy and the preservation of perfection. Science 279, 1210–1213. 10.1126/science.279.5354.1210 (doi:10.1126/science.279.5354.1210) 9469812

[75] Le Corre V Roux F Reboud X 2002 DNA polymorphism at the FRIGIDA gene in *Arabidopsis thaliana*: extensive nonsynonymous variation is consistent with local selection for flowering time. Mol. Biol. Evol. 19, 1261–1271. 10.1093/oxfordjournals.molbev.a004187 (doi:10.1093/oxfordjournals.molbev.a004187) 12140238

[76] Toomajian C 2006 A nonparametric test reveals selection for rapid flowering in the *Arabidopsis* genome. PLoS Biol. 4, 732–738. 10.1371/journal.pbio.0040137 (doi:10.1371/journal.pbio.0040137) PMC1440937 16623598

